# Analyzing crises in global financial indices using Recurrent Neural Network based Autoencoder

**DOI:** 10.1371/journal.pone.0326947

**Published:** 2025-07-14

**Authors:** Mimusa Azim Mim, Md. Kamrul Hasan Tuhin, Ashadun Nobi

**Affiliations:** 1 Department of Computer Science and Telecommunication Engineering, Noakhali Science and Technology University, Noakhali, Bangladesh; 2 Department of Computer Science and Engineering, Kishoreganj University, Kishoreganj, Bangladesh; University of Almeria: Universidad de Almeria, SPAIN

## Abstract

In this study, we present a novel approach to analyzing financial crises of the global stock market by leveraging a modified Autoencoder model based on Recurrent Neural Network (RNN-AE). We analyze time series data from 24 global stock markets between 2007 and 2024, covering multiple financial crises, including the Global Financial Crisis (GFC), the European Sovereign Debt Crisis (ESD), and the COVID-19 pandemic. By training the RNN-AE with normalized stock returns, we derive correlations embedded in the model’s weight matrices. To explore the network structure, we construct threshold networks based on the middle-layer weights for each year and examine key topological metrics, such as entropy, average clustering coefficient, and average shortest path length, providing new insights into the dynamic evolution of global stock market interconnections. Our method effectively captures the major financial crises. Our analysis indicates that interactions among American indices were significantly higher during the GFC in 2008 and the COVID-19 pandemic in 2020. In contrast, interactions among European indices were more prominent during the 2022 Russia-Ukraine conflict. In examining net inter-continental interactions, the influence was stronger between Europe and America during the GFC and the ESD crisis while, the influence between America and Asia was more powerful during the COVID-19 pandemic. Finally, we determine the structural entropy of the constructed networks, which effectively monitors the states of the market. Overall, our RNN-AE based network construction method provides valuable insights into market dynamic and uncovering financial crises, offering a powerful tool for investors and policymakers.

## 1 Introduction

The financial market is a dynamic, complex system where securities, commodities, and various assets are traded, often at prices influenced by global market conditions. Technological advancements and globalization have intensified competition in the financial sector, making it essential to analyze market dynamics comprehensively. Financial markets, characterized by their nonlinear, chaotic, and non-stationary nature, present significant challenges for predictive modeling and analysis. The non-stationary nature of financial time series implies that their statistical characteristics, such as the mean and variance, are not consistent over time. This variability introduces challenges in identifying persistent trends and stable correlations, as the underlying data patterns are subject to dynamic shifts [1,2]. As a result, analyzing stock markets to identify patterns, forecast future trends, and understand correlations between global indices is a longstanding area of research interest.

Recent research in this field has focused on using machine learning and RNN based deep learning models to improve stock market predictions by tracking historical data, recognizing patterns, and generating insights for buying and selling decisions. Among these models, Long Short-Term Memory (LSTM) networks, a type of RNN, have shown considerable success due to their ability to capture long-term dependencies in sequential data. However, despite their effectiveness in prediction, LSTM has received less attention for identifying correlations and constructing network structures from time-series data. While LSTM excels in time-series forecasting [3–5], autoencoders, known for automatically extracting features from input data, have also gained popularity for time series analysis [6]. Both LSTM and Gated Recurrent Unit (GRU) models are prominent in financial time series analysis and have been widely utilized in numerous studies. These models are often combined with Convolutional Neural Networks (CNNs) and attention mechanisms to further enhance prediction accuracy, and are applied across various time scales including daily, weekly, and monthly price data [7]. Furthermore, traditional approaches for constructing stock networks have largely relied on statistical techniques like cross-correlation, which may overlook the more complex, nonlinear relationships inherent in financial data.

In recent decades, researchers have explored numerous approaches to model the relationships between stock indices, aiming to capture their interdependencies and dynamics. Statistical techniques such as cross-correlation have been widely utilized to measure the strength and direction of linear relationships. For instance, Random Matrix Theory (RMT) and network techniques have been applied to investigate the correlation and network properties of twenty different financial indices [8]. China’s stock network structure and stability have been analyzed through threshold-based correlations [9], and U.S. stock closing prices from 2005 to 2009 were examined using complex networks [10]. To evaluate the impact of network size on its properties, financial networks are constructed for the Indian and Korean stock markets using correlation and thresholding methods [11]. Additionally, an LSTM-RGCN model was proposed that employs a correlation matrix to identify connections between stocks, effectively predicting stock market trends and outperforming the baseline model [12]. Correlation coefficients derived from the logarithmic returns of historical prices for Korean stock indices are used to construct complex networks, Minimum Spanning Trees (MSTs), and Hierarchical Networks (HNs), facilitating analysis of market conditions before, during, and after the global financial crisis [13]. As network-based methods like MST and Planar Maximally Filtered Graph (PMFG) gain popularity in global financial analysis, they analyze correlation networks to understand structural changes in the global market, often in response to financial crises [14–18]. However, traditional methods like MST and PMFG have limitations, as they exclude some significant relationships. To address these limitations, threshold networks offer alternative approaches, though challenges remain in defining effective correlation thresholds accurately [19–21].

Transfer entropy has been used to construct complex networks in financial markets, uncovering information flows and non-linear interactions during major market drops. It helps analyze the relationship between market and credit risk across economic periods [22–25]. Granger causality networks reveal the interdependencies among financial institutions, stock markets, and macroeconomic factors, showing causal relationships and varying connections across time frames [26–29]. Copula models have been used to examine financial networks and systemic risk, applied to U.S., Chinese, and global markets [30–32].

Numerous studies have employed network construction methods based on mutual information to account for the non-linear characteristics observed in real-world data. Both mutual information and its dynamic variant, the mutual information rate, have been applied within hierarchical networks [33]. In the field of multivariate time series analysis, a new method based on mutual-information matrices has been employed to explore nonlinear interactions, proving more effective than approaches relying on RMT [34]. A path-consistency algorithm combined with partial mutual information has been employed to construct stock networks for the Australian market, resulting in large clusters that correspond to various industrial sectors [35].

More recently, machine learning methods, particularly neural networks, have been applied to financial time series to enhance prediction accuracy and decision-making. Neural networks are favored for their data-driven nature and ability to model complex, nonlinear relationships, which traditional statistical models often struggle with [36–38]. A small number of recent studies have explored the use of machine learning techniques to build stock networks based on financial data. One such study applies feature ranking within a machine learning framework to assess the feature rankings and network characteristics of 21 different global stock indices [39]. Another study [40] examined the S&P 500 financial indices using a feature ranking approach, where the returns of stocks on one day were used to predict the feature rankings of the following day. These methods, however, primarily focus on feature ranking rather than network structure construction, leaving a gap in methods that use machine learning to dynamically capture and visualize relationships among global stock indices. Additionally, financial networks are constructed using random forests applied to high-frequency intraday data, revealing stronger connectivity and information flows among firms, as well as a higher network density before the 2007–2009 financial crisis, with larger firms showing better predictive power [41]. Deep learning networks, unlike traditional machine learning techniques, offer the advantage of automatic feature extraction, eliminating the need for prior manual selection of input features [42]. Furthermore, ensemble frameworks that incorporate BiLSTM with models like XGBoost and LightGBM have demonstrated high versatility and predictive performance in financial modeling tasks [43].

Traditional methods have inherent drawbacks, such as the inability of Pearson correlation to identify non-linear relationships between stocks, and the challenges that mutual information faces in handling multivariate correlations. Additionally, networks formed from mutual information and correlation lack directional properties, making it difficult to identify reciprocal influences among stocks. Standard Granger causality is constrained to linear systems and is highly sensitive to noise, while Dynamic Causal Modeling can address some non-linear interactions but necessitates prior knowledge of the network, making it unsuitable for exploratory research [44]. In feature ranking techniques, conventional machine learning algorithms like random forest and gradient boosting are often used to discover relationships between stocks. However, these models struggle with time series data because they generally assume that data instances are independent and identically distributed [45,46]. There has been limited exploration of alternative methods for identifying connections between stocks, as traditional approaches dominate most research. This study introduces a novel approach to building stock correlations from financial time series data to develop stock networks using a modified Autoencoder model RNN-AE.

This study distinguishes itself by applying an RNN-AE model to construct a threshold network from global stock market data. While RNN networks are commonly used in time-series forecasting and Natural Language Processing (NLP), their application in identifying stock index correlations remains underexplored [47,48]. The paper proposes an RNN-AE-based framework to identify intricate relationships across stock markets, offering a novel approach for capturing and visualizing the network structures that characterize global financial interactions. By doing so, the study aims to address a critical research gap and contribute to the understanding of how stock networks evolve, particularly during times of financial crisis. An RNN Autoencoder is a specialized architecture within the family of recurrent neural networks, incorporating variants such as basic RNNs, GRUs, and LSTM networks. It is designed to serve as an effective approach for unsupervised learning tasks, particularly in scenarios involving sequential data. It is used in various tasks related to reducing dimensionality, such as identifying anomalies, compressing data, and extracting relevant features [6,49,50]. This study aims to fill the limitations of existing network construction methods by introducing an RNN-AE model to capture and analyze yearly correlations between stock indices across 24 major global markets, utilizing daily closing prices and a one-year time window to segment the data. The RNN-AE model, consisting of multiple RNN (LSTM/GRU) encoder layers, a fully connected middle layer, and multiple RNN (LSTM/GRU) decoder layers, is trained on normalized daily returns data. The encoder processes input data sequentially, capturing temporal correlations and patterns, while the decoder reconstructs the encoded data. By utilizing the weights of the fully connected middle layer of the model, this study dynamically extracts correlations among stocks and constructs a threshold network to effectively visualize and analyze the relationships between various stock indices. Specifically, this paper seeks to analyze the structure of global stock market interactions, observe the influences of key markets, and assess how these structures respond to significant financial crises. Additionally, the approach aims to identify influential countries during periods of economic instability, providing insights into the dynamic behavior of global financial markets. The primary contributions of this paper can be summarized as follows:

- Introducing a novel RNN-AE-based approach to construct stock networks and extract local interactions across global indices.
- Observing the influences among 24 significant global stock markets.
- Analyzing how the local interaction structure changes dramatically during global financial bubbles or crises that occurred at different times, using threshold networks constructed from trained RNN-AE models.
- Identifying influential countries and their interactions with other countries during different crisis periods.

The remainder of this paper is organized as follows: Section 2 discusses the data and methodology, including details of the RNN-AE model. Section 3 presents empirical results, analyzing the network structures derived from our model. Finally, Section 4 concludes with key findings and implications of this work.

## 2 Data and methodology

### 2.1 Data description

In this research, we analyze the daily closing prices of the 24 stock indices representing the largest stock markets worldwide, which account for 96.6% of total stock market capitalization [51]. These stock markets are divided into three groups based on their geographical locations: Asia-Pacific, Europe-Africa, and America, as shown in Table 1. Countries are represented by their large-cap indices, which track prominent, high-capitalization companies and are widely regarded as indicators of overall market performance and economic trends. These indices are selected for their stability, liquidity, and ability to offer reliable long-term data. The historical stock market data used in this study, covering the period from January 1, 2007, to December 31, 2024, were obtained from Yahoo Finance [52], a widely recognized source for such information. To ensure consistency in analyzing global stock indices over this period, certain days are excluded based on market activity and public holidays. Specifically, if 30% or more of markets are closed on a given day, that day is excluded from the analysis. Conversely, if fewer than 30% are inactive, the last closing prices of the inactive markets are used to maintain a consistent dataset [53,54]. The dataset used in this study is available as Supporting Information (S1 Dataset).

**Table 1:** Details of the 24 countries and their stock indices.

Continent	Country (Code)	Index
**Asia-Pacific**	Australia (AUS)	S&P/ASX 200
	China (CHN)	SSE Composite
	Hong Kong (HKG)	HANG SENG
	India (IND)	NIFTY 50
	Japan (JPN)	Nikkei 225
	Korea (KOR)	KOSPI Composite
	Malaysia (MYS)	KLCI
	New Zealand (NZL)	S&P/NZX 50
	Singapore (SGP)	STI Index
	Taiwan (TWN)	Taiwan Weighted
	Thailand (THA)	SET
**Europe-Africa**	Belgium (BEL)	BEL 20
	France (FRA)	CAC 40
	Germany (GER)	DAX PERFORMANCE
	Italy (ITA)	FTSE MIB
	Netherlands (NLD)	AEX
	South Africa (SA)	JSE Limited
	Spain (SPA)	IBEX 35
	Switzerland (SWI)	SMI PR
	United Kingdom (UK)	FTSE 100
**America**	Brazil (BRA)	Bovespa
	Canada (CAN)	S&P/TSX
	Mexico (MEX)	Mexico S&P/BMV IPC
	United States (US)	S&P 500

### 2.2 Calculation of normalized return

The log returns of the daily closing prices are calculated by first computing the natural logarithm of the daily closing prices and then subtracting the previous day’s result from the current day’s result. The log returns over a time interval **∆t** is defined as:


$${rt}_{i}(t)=ln[p_{i}(t)-ln[p_{i}(t-\Delta t)]$$ (1)

where $p_{i}(t)$ represents the closing price of stock i on day t, and ${rt}_{i}(t$ denotes the log return on that stock on day t. For daily data, we typically assumed *∆t = 1*.

To ensure consistency and comparability across different stocks and markets, stock returns are normalized, eliminating scale differences and improving model convergence and performance. The normalization is carried out as follows:


$$r_{i}(t)=\frac{{rt}_{i}(t)-\left\langle {rt}_{i}(t)\right\rangle }{\sigma_{i}}$$ (2)

where $r_{i}(t)$ represents the normalized returns of stock *i* at time *t*, and <·> represents the mean over a one-year timeframe and $\sigma_{i}$ represen*t*s the standard deviation of the stock i over a one-year timeframe.

To mitigate potential biases introduced by extreme outliers, Winsorization is applied to the return data before normalization. Winsorization limits extreme values in both tails of the distribution, reducing the impact of rare, extreme fluctuations while preserving the overall data structure. In this study, a 0.5% Winsorization is applied to both tails, ensuring that extreme observations do not disproportionately influence the model’s learning process. This approach enhances the robustness of the model while maintaining meaningful variations in stock returns.

**Fig 1:**
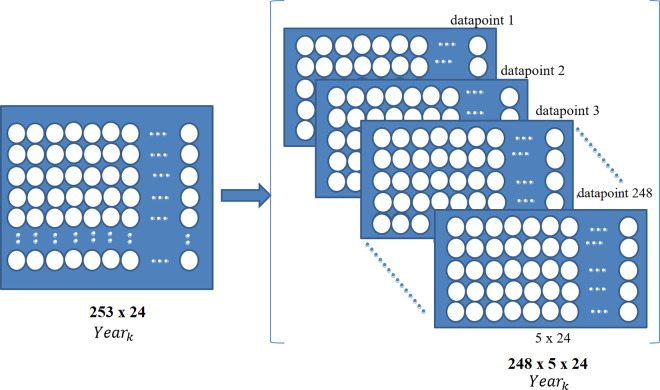
Dataset preparation from the stock returns in year *k* for model training.

The dataset includes a total of 4572 trade days, spanning the aforementioned duration. To facilitate analysis, we divide this dataset into one-year intervals, with 253 trading days each year. We examine a sliding window of varying sizes, ranging from 2 to 10, applied along the trading day dimension of the dataset with a step size of one. Our analysis reveals that a window size of 5 produces the most optimal results in terms of model performance. As the window shifts by one trading day at a time, it generates a total of 248 data points per year. Each data point represents a snapshot of the past five consecutive trading days, effectively capturing patterns and trends over that period. The study utilizes daily closing price data over a span of 18 years, from 2007 to 2024, for 24 predominantly traded countries. If we denote the prepared data of year *k* as *X*_*k*_ then *X*_*k*_* ∈ R* ^*P×T×N*^, where *P* is the total number of data points, *T* is the number of timestamps, and *N* is the number of stocks denoted as nodes. We employed a sliding window approach, which ensures that all input sequences maintain a uniform length without requiring padding or truncation. The overall process is illustrated in Fig 1.

### 2.3 RNN-Autoencoder (RNN-AE)

**Fig 2:**
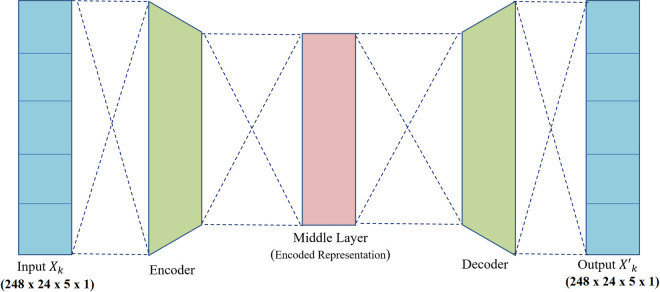
Architecture of the RNN-AE model.

Autoencoders are commonly utilized in representation learning to derive feature space representations of input data in an unsupervised manner. In this study, we employ a sequence-to-sequence autoencoder, tailored to our time-series dataset. The primary goal is to reconstruct the stock returns of the input using an encoded representation of the input sequence, while simultaneously uncovering relationships among stocks by identifying patterns and dependencies during the reconstruction process. An RNN-AE model consists of three main components: an initial set of RNN layers (LSTM/GRU) serving as encoder, a fully connected middle layer that captures latent correlations, and a final set of RNN layers (LSTM/GRU) functioning as decoders. The encoder sequentially processes the input data. At each time-step, each input component is analyzed, and the recurrent units learn to detect critical temporal relationships and patterns. The output of the encoder is an encoded representation of the input. This representation is passed to a fully connected layer, which is used to determine the learning correlations between the stocks. The encoded representations are then passed to the decoder. The decoder units are responsible for recovering the original input sequence from the encoded form. By training the RNN-AE on normalized returns for each individual year, we derive yearly correlation matrices between stock indices from the model weights (from 2D dense layer in the middle), with each matrix capturing the interrelationships specific to that year. The model captures the correlations of each stock by evaluating the influence of all other stocks on the target stock, thereby identifying their relationships with the target stock. Fig 2 illustrates the overall methodology employing RNN-AE. The model is implemented in Python 3 and evaluated on a local PC with the following specifications: CPU: Intel Core i5-10210U, RAM: 8 GB, GPU: Tesla T4 16GB GDDR6, and Operating System: Windows 10 Pro. Table 2 lists the layers and output forms of the RNN-AE model developed in Tensorflow 2.13.0. The model uses *N* TimeDistributed LSTM/GRU layers to encode the sequence, followed by a Dense 2D layer. The data is then reshaped and concatenated to maintain sequential structure. The second set of *N* TimeDistributed LSTM/GRU layers decodes the sequence, and a final TimeDistributed Dense layer reconstructs the output to the original shape, effectively capturing and reconstructing temporal patterns and highlighting stock correlations within the Dense 2D layer’s weights.

**Table 2:** RNN-AE model and its layers.

Layer (type)	Output Shape
*Input (Input Layer)*	*(None, 24, 5, 1)*
[time_distributed (LSTM/RGU)] *N	*(None, 24, hidden_size)*
*dense2d (Dense2D)*	*(None, 24, hidden_size)*
*expand_dims*	*(None, 24, 1, hidden_size)*
*split*	*(None, 24, 1, hidden_size)*
*concat*	*(None, 24, 5, hidden_size)*
[time_distributed (LSTM/GRU)] *N	*(None, 24, 5, hidden_size)*
*time_distributed(Dense)*	*(None, 24, 5, 1)*

An autoencoder consists of an encoder and a decoder. Let *F* be a feature space. An encoder is a function *φ: X → F* that learns the prominent characteristics and creates an encoded version of the sample in the feature space *F*. The encoded feature space *F* is then passed through a 2D dense layer, which is designed to learn inter-stock relationships by capturing interactions and dependencies between different features in the encoded representation. This layer enhances the model’s ability to extract meaningful patterns from the encoded data. The decoder is a function *ψ: F → X,* which aims to reconstruct the input using internal representation. Formally, given a sample input sequence $X_{k}$, the autoencoder is a function *Φ*_*AE*_*: φ ◦ ψ* that outputs ${X^{\prime }}_{k}$,


$$\phi AE(X_{k})={X^{\prime }}_{k}$$ (3)

### 2.4 RNN-AE model training and validation

The proposed autoencoder model is trained using unsupervised learning approach. Unsupervised learning is a type of machine learning in which a model identifies structures and patterns in data without using class labels or specific target outputs. If we identify the model in year *k* as “${RNNAE}_{k}$”, we may write it as follows when the model reconstructs the return sequence of stock *i* from the return sequences of all other stocks at time *t*,


$$\left(\begin{array}{c} r_{i}(t)\\ r_{i}(t-1)\\ \vdots \\ r_{i}(t-4) \end{array})={RNNAE}_{k}(\begin{array}{cccc} r_{1}(t) & r_{2}(t) & \cdots & r_{N}(t)\\ r_{1}(t-1) & r_{2}(t-1) & \cdots & r_{N}(t-1)\\ \vdots & \vdots & \ddots & \vdots \\ r_{1}(t-4) & r_{2}(t-4) & \cdots & r_{N}(t-4) \end{array}\right)$$ (4)

The model uses the return sequence of five consecutive trading days of stocks throughout the training and reconstruction phases, since the sequence length is set to five. Our model is trained for 25 epochs with a batch size of 16. In the experiments we perform, the activation function is Rectified Linear Unit (ReLU), and the Adam optimizer is used. For example, we calculate the reconstruction loss using the Mean Squared Error (MSE), which is calculated by first determining the squared difference between the reconstructed values and the original values at each time step and then taking the average of those squared differences. The reconstruction loss is calculated as follows:


$$ReconstructionLoss=\frac{1}{B*T*N}\sum_{t=1}^{B}\sum_{m=1}^{T}\sum_{n=1}^{N}{{|X^{\prime }}_{k_{tmn}}-X_{k_{tmn}}|}^{2},$$ (5)

where *T* represents the sequence length (5), *B* indicates the batch size (i.e., the number of data points in a particular batch), *N* denotes the total number of stock market, which is 24. ${X^{\prime }}_{k}$ represents the model’s reconstructed output in year *k*, and $X_{k}$ represents original input to the model in year *k*. In response to the reconstruction loss, which measures how well the model’s predictions match the original data, the model iteratively learns and improves by adjusting its parameters. Once the RNN-AE model is successfully trained, we extract the weight matrix from the fully connected layer positioned between the encoder and decoder. This weighted matrix or correlation matrix captures the relationships among the stocks and is denoted as *W*, with a shape of *N × N*.


$$W=[\begin{array}{ccc} W_{11} & \cdots & W_{1N}\\ \vdots & \ddots & \vdots \\ W_{N1} & \cdots & W_{NN} \end{array}]$$

It should be noted that this matrix is asymmetric, implying that the influence between any two stocks is not symmetrical. Consequently, each pair of stocks interacts with each other in a distinctive manner. Financial markets exemplify this behavior, as the magnitude of one stock’s influence over another differs from the reverse situation. Since we work with 24 stock indices, the shape of the matrix is 24 × 24. The value of the matrix *W,* associated with stocks *i* and *j*, can be used to determine the influence of node *i* on node *j*.

We assessed the ability of our model to capture the market’s underlying structure by validating it with network metrics. To optimize the model, we systematically tune hyper-parameters by conducting multiple experiments with varying configurations. The final selection was based on how well the model accurately represented the network structure and financial state. We evaluated various configurations of encoder and decoder layers (1, 2, and 3) to identify the ideal model depth that achieves a compromise between complexity and generalization. Multiple recurrent neural units, such as GRU and LSTM, were assessed to identify which most effectively captured sequential dependencies. The quantity of hidden units was altered among (32, 64, 128) to evaluate their influence on expressiveness and computing efficiency. Batch sizes of (8, 16, 32) were evaluated to assess their impact on training stability, convergence rate, and memory consumption. Furthermore, we examined various optimizers, such as Adam and SGD, and adjusted the learning rate within (1e-3, 1e-4, 1e-5) to guarantee smooth and stable training. These experiments helped in selecting the most effective configuration for our model.

### 2.5 Threshold network construction

The construction of threshold networks, in which a threshold value is assigned, is a common approach in financial network analysis [17,55–58]. After training the RNN-AE model, the weight matrix from the fully connected layer is used as the correlation matrix, representing the interaction between different stock indices. These weight matrix values indicate the strength of associations between stock indices. The nodes in a financial network represent individual financial entities, such as countries, while the edges capture the relationships between them. The interaction value between two indices determines the strength of their relationship, and the threshold value establishes whether a link between two nodes is significant.

To construct threshold networks, a fixed threshold (θ) is set at **0.0089**, ensuring a balanced network structure. This threshold is carefully selected to maintain full network connectivity while avoiding excessive edges that obscure meaningful financial relationships. If the threshold value exceeds 0.0089, the network becomes disconnected or fragmented, preventing effective analysis of inter-stock dependencies. Conversely, setting the threshold too low leads to an overly dense network with an excessive number of edges, making it difficult to extract meaningful structural insights. By choosing 0.0089, the network remains fully connected, preserving essential financial relationships without unnecessary noise.

In the constructed threshold network, if the interaction value between two stock indices exceeds the threshold, the corresponding element in the adjacent matrix is set to 1, indicating the presence of a link. Otherwise, it is set to 0, signifying no significant relationship. Once the threshold is determined, the network structure is established accordingly. The link between nodes *i* and *j* is added to the threshold network if the weight $W_{ij}$ between them is greater than or equal to the threshold, i.e., ${|W}_{ij}|\geq \theta$. Denoting the adjacency matrix of the threshold network by Adj, we can write:


$${Adj}_{ij}=\left\{ \begin{array}{c} {|W}_{ij}|,if{|W}_{ij}|\geq \theta \\ 0,otherwise \end{array}\right.$$ (6)

The weight matrix captures the influence of countries on stock values by assigning weights during the learning process. The feature matrix proposed in [40], referred to as the influence matrix, utilizes machine learning methods to estimate the probability of interactions between stock indices. In contrast, the weight matrix in this study captures both positive and negative correlations, reflecting opposing and supportive influences. This provides deeper insights into global market dynamics, particularly in understanding how financial crises impact stock market inter-dependence.

## 3 Results and discussions

We obtain results after training the model using the following parameter setup. The optimal design includes a single encoder and decoder layer, both implemented with LSTM. Each layer is set with 64 hidden units to maintain consistency. Middle layer weights are initialized with a normal distribution using a random seed of 1. The model is trained for 25 iterations using the Adam optimizer with a learning rate of 1e-4 and a batch size of 16. MSE is used as the evaluation metric to ensure effective optimization and precise performance monitoring.

### 3.1 Latent correlation from model’s weights

**Fig 3:**
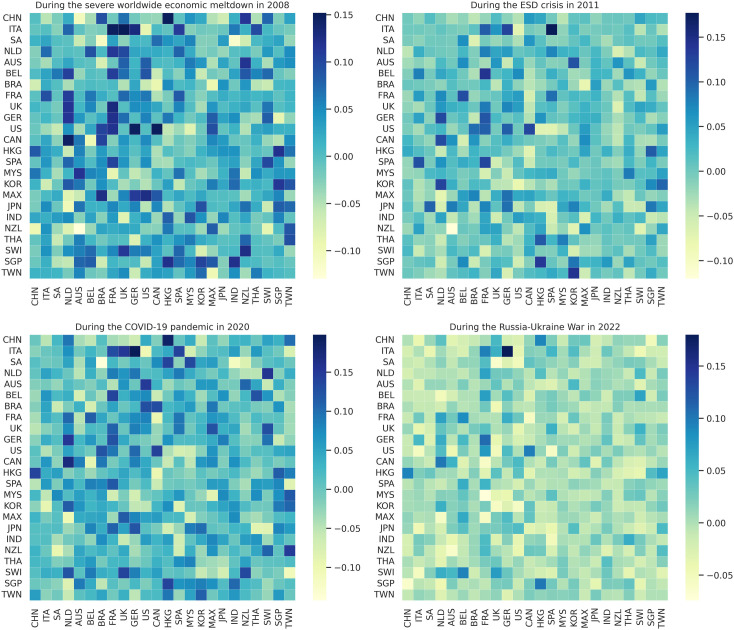
Heatmaps of weight matrices for all stocks during four different time periods: (a) during the severe worldwide economic meltdown in 2008, (b) during the ESD crisis in 2011, (c) during the COVID-19 pandemic in 2020, and (d) during the Russia-Ukraine War in 2022. A darker color indicates a higher influence, and a lighter color specifies the opposite. The influence of countries during the severe worldwide economic meltdown (2008) and the COVID-19 pandemic (2020) was higher than during other crises.

After training the model, we extract the weights from the middle layer to serve as a latent correlation matrix among stocks, as this layer captures the interdependencies between various indices. The weight matrix is asymmetrical, indicating that developed markets tend to exert greater influence on developing or emerging markets, while the reverse influence maybe minimal or absent. Fig 3 illustrates the heatmaps of weight matrices for all stocks during four major crises: the 2008 GFC, the 2011 ESD crisis, the 2020 COVID-19 pandemic, and the 2022 Russia-Ukraine War. The matrices, representing 24 stock indices, assess the influence of stock *i* on stock *j*. Each row in W corresponds to a specific stock, with the values indicating the contribution of other stocks to the model’s prediction for that stock. The greater the value, the more significant the influence. The lighter colors in Fig 3 indicate a stronger negative stock influence, while darker colors indicate a stronger positive stock influence.

Since the Great Depression, countries have played a crucial role during major global economic crises, particularly the 2008 financial crisis. Fig 3a illustrates strong correlations among countries in the Americas, Europe-Africa, and the Asia-Pacific region. Particularly, countries like United States, Canada, Germany, and Italy exhibit noticeably strong positive influences on other countries, as indicated by the darker shades. On the other hand, countries like South Africa, China, and India shown noticeable negative influences on certain regions, reflected by the lighter shades. This period reflects a time of high global interdependence, where shocks in one market rapidly propagated worldwide. The 2007–2008 financial crisis, which began in the U.S. subprime mortgage market, triggered widespread market turmoil, significantly affecting developed and emerging economies alike. The early stages of the crisis began in the second half of 2007 and peaked in September 2008. Influence remained elevated, particularly among most European and some Asian and American countries, due to the 2011 European Sovereign Debt Crisis, as depicted in Fig 3b. The heatmap reveals strong regional interconnectedness in Europe, with countries such as Italy, Germany, France, and Spain exerting significant positive influences on surrounding nations, indicating a high degree of financial stress propagation. In contrast, countries like Japan, Australia, and Mexico exhibited noticeable negative influences on specific markets, suggesting an inverse reaction to financial turbulence.

During the COVID-19 pandemic, the heatmap in Fig 3c illustrates widespread and relatively strong correlations across global markets. Financial interdependence remained significant, with both strong positive and negative influences observed across regions. Specifically, countries such as China, United States, Canada, United Kingdom, and Germany exhibit strong positive influences, while countries like Sweden, Malaysia, and Brazil demonstrate moderate negative influences. The pandemic-induced financial turmoil reflected a unique crisis pattern, where economic disruptions were not only regionally concentrated but also exhibited global spillover effects. Overall, advanced economies in North America, Europe, and the Asia-Pacific played pivotal roles in transmitting financial influence, with patterns of influence shifting depending on the type of crisis and geographic proximity. Finally, relatively weaker inter-country influence is observed during the Russia-Ukraine war, as shown in Fig 3d. This highlights geopolitical risks and economic instability, particularly stemming from energy dependencies, and points to a more localized impact. Notably, Italy, China, South Africa, and the Netherlands exhibit weak positive influences, while Thailand, Switzerland, Singapore, and Taiwan show weak negative influences.

### 3.2 Threshold networks

**Fig 4:**
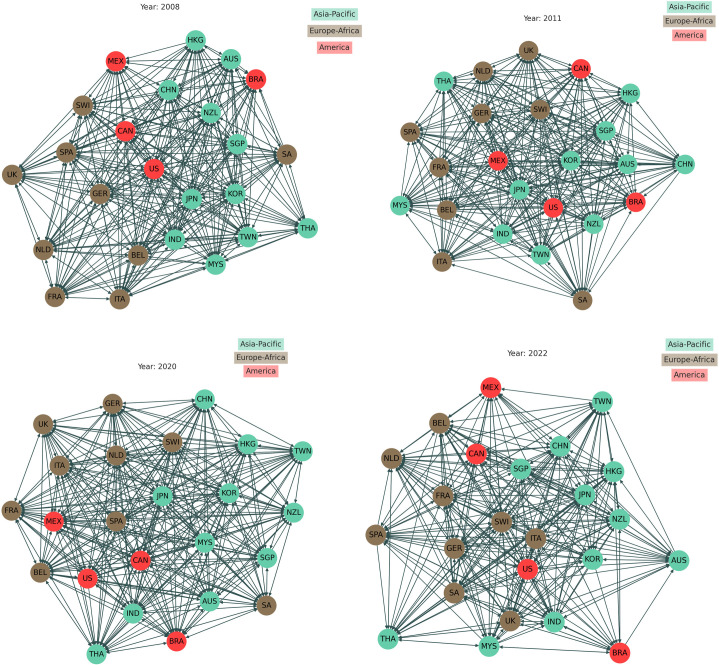
Visualization of the structure of the threshold network using the threshold of 0.0089 for the influential 24 countries across three continents during four distinct time periods: (a) during the severe GFC in 2008, (b) during the ESD crisis in 2011, (c) during the COVID-19 pandemic in 2020, and (d) during the Russia-Ukraine war in 2022. A higher out-degree for a node indicates that a country is more influential in the network. The influence of countries during the severe worldwide economic meltdown in 2008 and the COVID-19 pandemic in 2020 was significantly higher compared to other crises, reflecting heightened global financial interdependence and intensified international interactions during these periods.

Fig 4 depicts the threshold network for the 24 countries as a directed graph, where each node (*V*) represents a distinct index and each link (*E*) represents the connection between the two indices, which is weighted by the cross-correlation value of the return time series between the two indices.

In Fig 4a, during the GFC, countries such as the United States, Taiwan, and Korea exhibited the highest levels of interaction, reflected by their higher degree of connectivity within the network. The United States, for example, had an out-degree of 43 connections. In contrast, countries like Thailand and France were among the least interactive nodes due to their lower connectivity. The severity of the crisis was underscored by the network’s 462 edges, representing extensive international interactions. As shown in Fig 4b, the ESD crisis in 2011 saw a shift in the interaction landscape. The Netherlands and France had the highest degrees of connectivity, while India, China, Italy, and South Africa remained on the network’s periphery due to their lower connectivity. In 2020, during the COVID-19 pandemic (Fig 4c), Belgium and Germany emerged as the most interactive nodes, whereas South Africa, Australia, and the United States were positioned at the network’s periphery. This crisis had an even broader impact, demonstrated by 486 edges in the network, reflecting widespread international interactions. By 2022, during the Russia-Ukraine war (Fig 4d), overall influence slightly declined. Belgium and Germany were the most interactive countries, while South Africa, Australia, and the United States showed minimal interaction.

The high number of connections in the 2008 network (462) and the 2020 network (486) underscores the persistent interdependence of global economies. This interconnectedness facilitated the rapid transmission of financial shocks, amplifying the impact of market disruptions worldwide. These evolving patterns highlight the necessity for coordinated international financial policies to mitigate future crises. The vulnerabilities exposed during past financial crises have reinforced the need for strategic responses, prompting shifts in investor behavior. Countries like Germany and the United States continue to be perceived as stable investment destinations, significantly influencing global capital flows and financial stability.

### 3.3 Analyzing interactions within and between continents

We measure the average interactions within a continent by calculating the interactions between each pair of countries within that continent and then averaging these interactions, which provides a quantitative measure of intra-continental connectivity. This allows for a clearer understanding of regional financial dynamics and can be used to analyze the stability and influence of countries within specific continents, offering insights into how localized financial interactions evolve over time. We define the intra-continental interaction as [59]:


$${\overset{―}{I}}_{ij}^{in}(m)=\frac{1}{E}\sum_{i\neq j}W_{ij}(m)$$ (7)

where W_*ij*_*(m)* represents the cross-interaction between stock indices *i* and *j* in the same continent *m*, and *E* is the total number of such edges.

We also evaluate the influence between continents by examining the impact one continent has on another, using the weights assigned to the connections between nodes within the influencing continent and those within the affected continent. To quantify the influence of continent *m* on continent *n*, we sum the weights of the connections that originate from nodes in continent *m* and extend to nodes in continent *n*. Denoting the influence of continent *m* on continent *n* as ${\overset{―}{I}}_{ij}^{be}(mn)$, the inter-continental interaction can be expressed as follows:


$${\overset{―}{I}}_{ij}^{be}(mn)=\sum_{i\in m}\sum_{j\in n}W_{ij}$$ (8)

where i∈m denotes a country i within continent m, j∈n denotes a country j within continent n.

Following the calculation of the inter-continental influence matrix, we determine the net inter-continental interaction, which quantifies the net flow of information between continents. Net inter-continental interaction between continents m and n is defined as,


$${\overset{―}{NI}}_{ij}^{be}(mn)=\left\{ \begin{array}{c} abs({\overset{―}{I}}_{ij}^{be}\left(mn)\right)+abs({\overset{―}{I}}_{ij}^{be}(nm)),ifm\neq n\\ 0,oterwise \end{array}\right.$$ (9)

**Fig 5:**
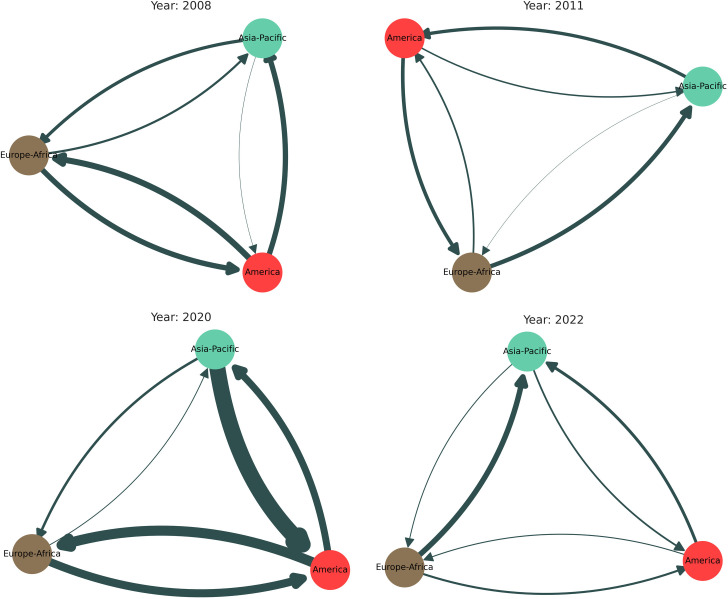
(*Inter-continental Interaction*) Visual representation of the interaction among three continents (Asia-Pacific, Europe-Africa, and America) during four different time periods: (a) during the worldwide financial meltdown in 2008, (b) during the ESD crisis in 2011, (c) during the COVID-19 pandemic in 2020, and (d) during the Russia-Ukraine War in 2022.

Fig 5 visually represents how different continents interacted with each other during specific times of crisis. These interactions are visualized as networks, where connections represent the strength of influence between regions, based on ${\overset{―}{I}}_{ij}^{be}(mn)$ matrices. As shown in Fig 5a, during the 2008 GFC, America was the most influential region, exerting the highest influence on Europe-Africa and the second highest on Asia-Pacific. This dominance underscores America’s central role in triggering and transmitting financial shocks globally during the crisis, particularly as it originated from the U.S. subprime mortgage market. Europe-Africa also had a strong influence on America. In contrast, the Asia-Pacific region exerted the least influence on others, indicating a more passive role in the global contagion at that time. During the COVID-19 pandemic, Fig 5c showed that America continued to exert substantial influence, particularly on Europe-Africa. Interestingly, the Asia-Pacific region demonstrated a strong and increased influence on America. However, the influence between Europe and Asia remained relatively weak, suggesting limited direct financial coordinated responses between these regions.

In the 2011 ESD crisis, Fig 5b demonstrated that Europe-Africa had a considerable impact on Asia-Pacific. Additionally, America exerted a moderate influence on Europe-Africa. Overall, the intensity of cross-regional influences during the ESD crisis was lower than that observed in the 2008 GFC and the 2020 COVID-19 pandemic, underscoring the more regionally contained nature of this event. This pattern highlights the ESD crisis as a primarily Europe-centered event with limited global contagion effects compared to broader systemic crises. Fig 5d shows that during the 2022 Russia-Ukraine war, Europe-Africa exerted moderate influence on the Asia-Pacific region, though overall interregional financial influence was lower than in previous crises. America’s role was less dominant, reflecting the conflict’s more localized economic impact, particularly in energy markets. The Asia-Pacific region remained largely passive, indicating limited global contagion. This suggests the war’s financial effects were more regionally contained compared to the broader systemic shocks of 2008 and 2020.

We observe how the local interaction structure changes dramatically during global financial crises and disruptions, as shown in Table 3. The table indicates that during the 2008 GFC, the 2011 ESD crisis, and the 2020 COVID-19 pandemic, America exhibited the highest average intra-continental interactions $(\overset{―}{I_{ij}^{in}}$), highlighting its dominant role in regional financial dynamics. However, during the Russia-Ukraine war in 2022, Europe-Africa displayed the highest intra-continental interactions, reflecting the region’s intensified internal financial activity amid geopolitical tensions.

**Table 3:** $\overset{―}{I_{ij}^{in}}$ and $\overset{―}{{NI}_{ij}^{be}}$ represent the interactions inside and between the continents (Asia-Pacific, Europe-Africa and America), respectively, for four major financial crises particularly in 2008, 2011, 2020, and 2022.

Year	$\overset{―}{I_{ij}^{in}}$			$\overset{―}{{NI}_{ij}^{be}}$		
	Asia-Pacific	Europe-Africa	America	Asia-Europe	Europe- America	America-Asia
**2008**	0.05249	0.06283	**0.09509**	0.25094	**0.527960**	0.262918
**2011**	0.03973	0.05528	**0.05838**	0.213223	**0.248966**	0.223961
**2020**	0.05170	0.06802	**0.08739**	0.156953	0.843761	**1.068612**
**2022**	0.02823	**0.04364**	0.04022	**0.295500**	0.143703	0.219574

The strong inter-continental interactions ($\overset{―}{{NI}_{ij}^{be}}$ between Asia-Pacific, Europe, and America suggest that economic shocks in one region can quickly propagate to others, intensifying global market volatility. Significant inter-continental interactions between Europe-Africa and America were observed during the 2008 GFC and during the 2011 ESD crisis. Notably, during the COVID-19 pandemic, the interaction between America and Asia-Pacific was the highest. In contrast, the interaction between Asia and Europe has consistently remained lower across all periods except the Russia-Ukraine War. From an investment perspective, these results emphasize the importance of global diversification, as financial markets are highly interdependent. Economies that are geographically distant can still have a profound influence on each other, meaning that financial crises tend to ripple across continents, affecting all regions. Understanding these inter-continental linkages is crucial for investors, policymakers, and institutions seeking to navigate global financial turmoil.

### 3.4 Network properties

**Fig 6:**
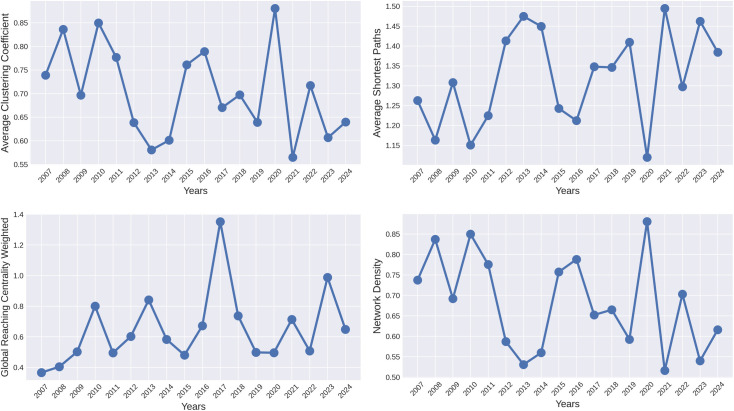
Topological properties of threshold networks of the top 24 indices over the time span from 2007 to 2024, using the fixed threshold approach with a threshold value of 0.0089: (a) Average Clustering Coefficients, (b) Average Shortest Path Lengths, (c) Weighted Global Reaching Centrality, and (d) Network Density. The GFC of 2008 and COVID-19 pandemic of 2020 were the most influential and biggest financial crises of the 21st century.

Let’s consider topological changes in the global financial networks produced by the weight matrices during different years. In a financial network of size *N*, the countries are represented as nodes, and the connections between them, or edges, are determined based on a predetermined threshold. An analysis of the global financial network is presented in Figs 6a,6d. These Figs illustrate the average clustering coefficient, average shortest path length, global reaching centrality, and network density.

#### 3.4.1 Average clustering coefficient.

**Fig 7:**
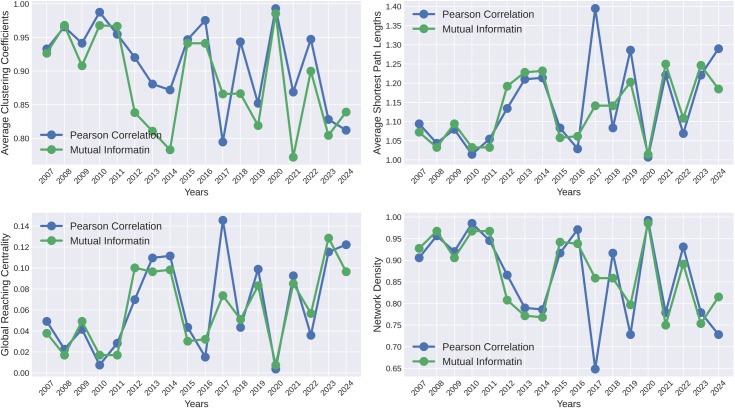
The topological properties of threshold networks for the top 24 indices are analyzed over the period from 2007 to 2024, utilizing two widely used network construction methods: Pearson Correlation and Mutual Information. In both cases, the threshold is defined by the mean values of the corresponding matrices. (a) Average Clustering Coefficients, (b) Average Shortest Path Lengths, (c) Global Reaching Centrality, and (d) Network Density.

The average clustering coefficient evaluates how frequently nodes in a network form clusters or groups of interconnected neighbors. The average clustering coefficient of a network *G* can be defined as [60,61],


$$C=\frac{1}{N}\sum_{i=1}^{N}\frac{m_{i}}{n_{i}(n_{i}-1)}$$ (10)

where, *m*_*i*_ is the number of edges connecting the neighbors of node *i*, and *n*_*i*_ is the number of neighbors of node *i*. If $n_{i}<2,$ the clustering coefficient of node i is assumed to be zero. Fig 6a illustrates the average clustering coefficients of constructed networks over different periods, showing notable peaks during major crises. These findings reveal that during severe global financial crises, such as the 2008 GFC, and the COVID-19 pandemic, the average clustering coefficient reaches its highest levels. Higher clustering coefficients were also observed during other financial crises, including the ESD crisis (2010-11), and the 2022 Russia-Ukraine war. Elevated clustering coefficients suggest that financial markets become more interconnected, with countries exerting greater influence on each other, increasing the likelihood of contagion. In contrast, during periods of market stability, such as 2021, when global equities performed strongly, the average clustering coefficient dropped sharply, reflecting reduced interdependencies. This pattern underscores that during times of economic turmoil, financial markets become more sensitive to shocks, highlighting the importance of monitoring network properties for effective risk assessment and crisis management. Fig 7 presents the topological properties of financial networks constructed using two widely employed methods: Pearson Correlation and Mutual Information. As illustrated in Fig 7a, both methods exhibit high clustering coefficients during periods of crisis and low clustering coefficients during periods of stability. This observation aligns with findings from previous research [39], which demonstrated similar trends in clustering coefficients for feature-ranking-based network constructions during crisis and non-crisis periods. While comparing the average clustering coefficients across different network construction methods, our proposed approach exhibited the same pattern, albeit with greater clarity and a more pronounced definition.

#### 3.4.2 Average shortest path.

The average shortest path length, also known as the characteristic path length, is the average number of edges required to connect any pair of elements in a network or a graph. It quantifies the overall efficiency of communication within a network. The average shortest path length of a connected network can be written as [53,61,62],


$$a=\sum_{s,t\in V}\frac{d(s,t)}{N(N-1)}$$ (11)

where *V* is the set of nodes in a network *G*, (s, t) is the shortest path from node *s* to node *t*, and *N* is the number of nodes in *G*. As shown in Fig 6b, during different financial crises, such as those in 2008, 2011, 2020, and 2022, the average shortest path lengths decrease significantly, indicating a greater degree of interdependence between stock indices. This drop in path lengths reflects a more tightly connected network where financial shocks can propagate more rapidly and extensively across different markets. In periods of crisis, the increased connectivity suggests that disruptions in one region are more likely to affect others, amplifying the risk of contagion. Conversely, during stable periods, average shortest path lengths are higher, signifying a more loosely connected network where indices exhibit reduced interdependence, limiting the spread of financial shocks. These fluctuations in path lengths underscore the evolving structure of financial networks and emphasize how crises heighten market sensitivity, necessitating vigilant monitoring to manage potential risks effectively. A similar trend is evident in other network construction methods, as shown in Fig 7b and in [39,53]. However, our method captures this pattern with greater clarity, particularly during crisis periods, and provides a more comprehensive understanding of the underlying network dynamics.

#### 3.4.3 Global reaching centrality.

Global Reaching Centrality (GRC) is a global network quantity that calculates the hierarchy flow of a complex network. A network’s GRC can be defined as the mean value derived by subtracting the local reaching centrality of each node from the greatest local reaching centrality observed among all nodes in the network. It measures the overall significance of a node based on its capacity to communicate with other nodes in the network. It can be defined as [63],


$$GRC=\frac{\sum_{i\in V}\left[C^{\max}-C(i)\right]}{N-1}$$ (12)

where *C(i)* is the Local Reaching Centrality (LRC) of node *i*, and *C*^*max*^ is the maximum value of LRC. Fig 6c depicts the evolution of global reaching centrality in the weighted threshold networks over time. The curve of global reaching centrality from 2007 to 2024 illustrates notable variations influenced by key financial events and crises. Significant declines in global reaching centrality are observed during periods of severe economic turmoil, such as the 2008 GFC, the 2011 ESD crisis, the 2020 COVID-19 pandemic, and the 2022 Russia-Ukraine war. These drops indicate a reduction in the influence of major stock indices, reflecting less hierarchical financial markets during crises. Conversely, periods of recovery and market stability, particularly between 2009-10, 2012-13, and 2016-17, show marked increases in global reaching centrality, signaling greater influence of dominant indices on the global financial network. The peaks in 2017 and 2023, for instance, suggest a period of robust market performance and stability. High global reaching centrality values are also observed during crises in Pearson Correlation and Mutual Information based methods illustrated in Fig 7c. Notably, the curve generated by our method demonstrates a greater ability to accurately identify crises, offering enhanced precision in detecting these periods.

#### 3.4.4 Network density.

Network density is the ratio of the number of existing links to the maximum number of possible links. A higher-density network implies a more interconnected network, whereas a lower-density network denotes a sparser or less-connected network. The density of a network *G* can be expressed as [64],


$$d=\frac{m}{N(N-1)}$$ (13)

where *m* represents the number of edges and *N* represents the number of nodes in *G*. Fig 6d illustrates the Network Density curve for threshold networks over the period 2007-2024. This curve exhibits notable fluctuations, indicating varying levels of connectivity among stock indices during different time frames. Peaks in density are observed in 2008, 2010-11, 2020, and 2022 corresponding to major global crises such as the 2008 GFC, the European Debt Crisis, the COVID-19 pandemic, and the Russia-Ukraine conflict. During these periods, higher network density (close to 0.9 in most cases) indicates increased interconnections and interdependencies, which reflect a heightened risk of contagion across financial markets. Conversely, sharp declines in density occur during relatively stable periods, such as 2013, 2019, 2021, and 2023 where the values dip as low as 0.5-0.6, suggesting fewer interdependencies and a more fragmented network structure. These variations highlight how financial markets become more tightly interconnected during crises, potentially increasing systemic risks, while during stable periods, the connectivity between indices is reduced. Our results on network density are consistent with our methodology and the findings of earlier studies [39,53]. Furthermore, our approach consistently produces superior curves, showcasing reliable performance across various network building techniques, including those illustrated in Fig 7d.

#### 3.4.5 Structural entropy.

Entropy is the measurement of the uncertainty in a network’s arrangement of nodes and edges. To compute entropy, we must initially determine the degree of each node. The probability of each node is then calculated by dividing its degree by the total degree of all the nodes in the threshold network.


$$p_{i}=\frac{deg(i)}{\sum_{i=1}^{N}deg(i)}$$ (14)

where $p_{i}$ denotes the probability of node i and deg(i) represents the degree (in-degree + out-degree) of node i. Because the total probability of all countries is one, we can calculate the entropy to understand the state of the market. The entropy of the whole network is calculated as [65],


$$S=-\sum_{i=1}^{N}P_{i}*\log_{2}(P_{i})$$ (15)

To define the entropy within a continent as,


$$S^{in}(m)=-\sum_{i=1}^{N_{m}}P_{i}*\log_{2}(P_{i})$$ (16)

where $P_{i}$ represents the probability of stock index *i* in the continent *m* and $N_{m}$ is the total number of nodes in the continent.

**Fig 8:**
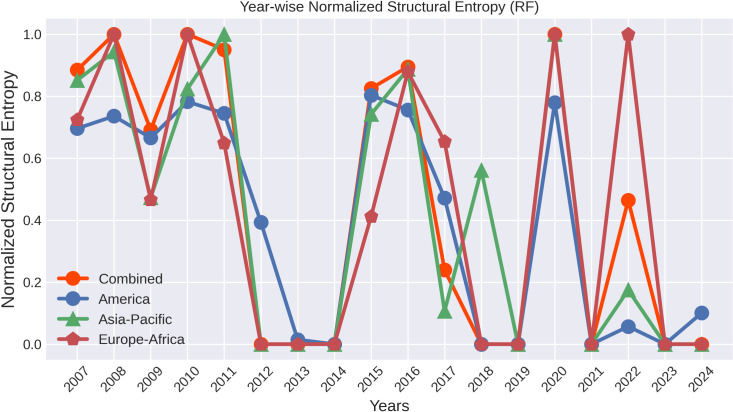
The variation in structural entropy over time within the countries for the three specified continents, and overall global trends. Higher entropy indicates a more uniform distribution of influence among the countries.

**Fig 9:**
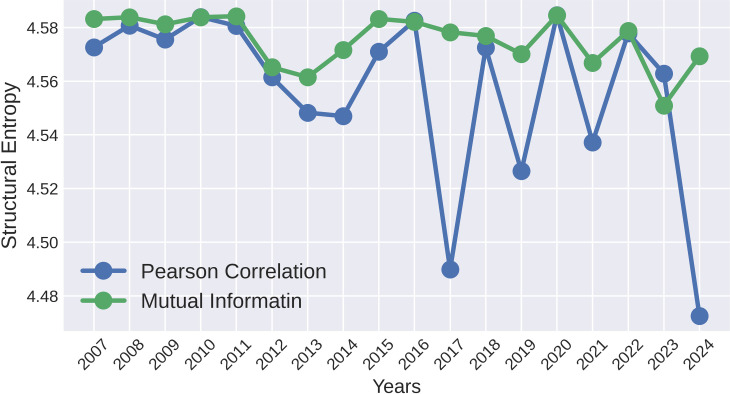
Structural entropy of threshold networks for the top 24 indices are analyzed over the period from 2007 to 2024, utilizing two widely used network construction methods: Pearson Correlation and Mutual Information. In both cases, the threshold is defined by the mean values of the corresponding matrices.

Fig 8 illustrates significant fluctuations in normalized structural entropy across America, Asia-Pacific, and Europe-Africa, and overall global entropy from 2007 to 2024, highlights rising entropy levels during significant global financial crises. By normalizing the entropy values, the Fig enables a consistent comparison across continents, represented through four distinct line graphs. The graph illustrates the Normalized Structural Entropy for networks in the America, Asia-Pacific, and Europe-Africa regions, along with the Overall Entropy from 2007 to 2024. The entropy measures the level of disorder within these networks, where higher values indicate more balanced and distributed influence, and lower values signify increased concentration or centralization. During major financial crises like the 2008 GFC and the 2020 COVID-19 pandemic, all three regions, as well as the overall network, exhibit sharp peaks in entropy, reflecting a more complex, less predictable market and interconnected distribution of influence. Conversely, during periods of stability, such as 2012-14, 2018-19, and 2023-24, the entropy drops significantly, indicating a more centralized network structure dominated by key nodes. While all regions follow this general pattern, slight variations exist in the timing and magnitude of these changes, highlighting regional differences in how financial systems respond to global and local events. This dynamic nature of structural entropy underscores the importance of understanding network interconnectedness for assessing vulnerability and resilience in global financial markets. A similar pattern is observed for structural entropy in both the Pearson Correlation and Mutual Information-based methods depicted in Fig 9, where entropy levels are elevated during crisis periods and lower during non-crisis periods. By examining the curves across different methods, the pattern of entropy decline during a crisis is more clearly understood in the method we propose.

### 3.5 Comparative analysis

To strengthen the discussion, a comparative analysis with prior studies is incorporated to highlight the advantages of the RNN-AE model over traditional correlation-based methods and other machine learning techniques. Existing studies that utilize Pearson Correlation and Mutual Information for global financial network construction reveal distinct patterns in the topological properties of networks during crisis periods, which are consistent with our findings [8,17,53,66]. In addition, our approach has also been applied to local stock markets, yielding results that are consistent with those of existing studies [67]. However, these traditional methods are limited in capturing complex, non-linear dependencies, leading to less accurate market representations during volatile periods. Unlike Random Forest and gradient boosting models, which assume independent and identically distributed data and thus struggle with sequential dependencies, the RNN-AE model effectively captures temporal correlations and evolving relationships within stock indices.

Previous feature-ranking-based approaches [39,40] have attempted to refine market structure representation, but our model provides a more precise and dynamic structural representation of market interactions, particularly during crisis periods. Additionally, entropy-based methods, including transfer entropy, have been widely used to analyze financial risk [23–25]. However, these methods primarily focus on information flow rather than network topology, limiting their ability to capture the evolving structure of financial networks. Overall, our results demonstrate superior accuracy and robustness compared to existing approaches, particularly in crisis detection and structural analysis. By integrating deep learning into financial network construction, this study advances the field beyond previous methodologies and provides an effective tool for financial risk assessment. The RNN-AE enables the extraction of non-linear dependencies in time series data, often overlooked by traditional statistical or shallow learning techniques, allowing it to more effectively capture underlying latent structures. The results obtained from the topological analysis of the networks generated through our method show its efficiency.

During several crisis events like the 2008 GFC, the COVID-19 pandemic in 2020, and the 2022 Russia–Ukraine conflict, our approach consistently shows more pronounced spikes in average clustering coefficients and sharper decreases in average shortest path lengths compared to networks built using Pearson correlation, mutual information, and random forest [39,40,53,54]. In the same context, the global reaching centrality computed from our model displays clearer hierarchical changes during moments of turmoil, more accurately than traditional statistical and shallow machine learning approaches. The network density and structural entropy computed from our model are also more sensitive to systemic changes, and therefore these patterns become more useful in the tracking of contagion risks and market centralization.

However, the proposed method also has drawbacks. As a deep learning model, it requires a large amount of data for training and a computationally powerful device, limiting its utility for shorter time periods beyond one year and making it difficult to use in contexts with low computational resources or where rapid analysis is needed. RNN autoencoder models are challenging to interpret because of their deep, recurrent architecture and non-linear transformations, which obscure the learned relationships and features within the hidden layers. Additionally, tuning hyperparameters and ensuring convergence of training can be challenging and time-consuming, often requiring extensive experimentation and careful optimization.

## 4 Summary and concluding remarks

Crises in financial markets have gained increasing attention in recent years. In this study, we introduce an innovative approach for constructing stock networks using a deep learning model, RNN-AE. Our method effectively identifies financial crises by recognizing patterns and similarities with previous financial bubbles. By analyzing interactions among 24 global stock markets, we identify influential countries and examine their relationships with other countries. This represents a significant shift from traditional approaches, such as linear and nonlinear correlation techniques and earlier feature-ranking methods, by introducing a groundbreaking deep learning framework for network construction.

Our research focuses on examining how the local interaction structure changes dramatically during a global financial bubble using networks constructed from trained RNN-AE models. The analysis of global financial networks spanning 2007–2024 reveals topological characteristics that act as reliable indicators of impending crises, with distinct peaks coinciding with major economic downturns. Notably, our findings demonstrate distinct patterns of interdependence among global stock indices. During the 2008 GFC and the 2020 COVID-19 pandemic, American indices exhibited increased interactions, suggesting heightened regional interconnectivity. In contrast, the 2022 Russia-Ukraine conflict was characterized by more pronounced interactions among European indices, indicating a localized impact within the European market, while the 2011 ESD crisis exhibited moderate interactions among European indices. This is supported by existing studies such as [68], which observed pronounced regional effects within European markets during this period. These findings align with our results, where regional interdependence during these crises was notably concentrated within specific geographical boundaries, highlighting localized financial impacts.

Furthermore, intercontinental interactions offer valuable insights into systemic financial risks. During the GFC and the ESD crisis, the transatlantic link between Europe and America was the most pronounced. Meanwhile, the COVID-19 pandemic led to stronger financial linkages between America and Asia, underscoring regional variations in crisis impact. Our research also demonstrates the utility of structural entropy as a key metric for monitoring market stability. By analyzing entropy trends, we gain deeper insights into the evolving dynamics of financial systems and their susceptibility to crises.

The findings of this study have significant implications for policymakers, investors, and market regulators. By identifying key countries as central nodes within financial networks, our methodology provides a detailed country-wise analysis of stock market behaviors during crises. This knowledge can inform risk management strategies, policy interventions, and investment decisions to enhance market stability. Decision-makers must leverage these insights to implement effective measures that mitigate systemic risks and promote financial resilience.

### Future work

For future research, it would be valuable to investigate the underlying factors influencing entropy trends and threshold network behavior in global financial markets. Exploring correlations with economic indicators, geopolitical events, and market sentiment could provide a more comprehensive understanding of financial stability. Additionally, extending the dataset beyond 2024 will allow for better analysis of ongoing crises, such as the Russia-Ukraine conflict. Further research should also examine the implications of regional financial influences on global market stability. Understanding how crises affect different continents to varying degrees will enhance financial risk assessment and crisis mitigation strategies.

While this study demonstrates that a five-day trading window provides the most accurate and stable network structure, a more extensive analysis is needed to confirm its optimality. Future work will incorporate different window sizes, such as 10 and 15 days, to evaluate their impact on network behavior and result stability. Conducting a sensitivity analysis on varying window sizes will further validate the robustness of our findings and provide insights into the best time frames for capturing financial dependencies. Additionally, this study effectively captures short- to medium-term dependencies using a five-day sequence, but future work will focus on modeling long-term dependencies in financial data. Extending the sequence length beyond five days requires a larger dataset to ensure sufficient training data per year. Future studies will address this challenge by incorporating longer historical datasets, enabling better analysis of prolonged financial trends and structural changes in global markets.

Furthermore, the Autoencoder model used in this study requires large datasets for effective training, making it less suitable for shorter time frames with limited data. Its high computational cost and interpretability challenges also limit its application in real-time financial analysis. Future studies should explore alternative models that balance accuracy, efficiency, and interpretability for improved financial forecasting.

## Supporting information

S1 Dataset Daily closing prices of 24 global financial indices (2007–2024). This dataset was used for training and evaluating the RNN-based autoencoder model.
(CSV)
